# Binding of the Phage Display Derived Peptide CaIX-P1 on Human Colorectal Carcinoma Cells Correlates with the Expression of Carbonic Anhydrase IX

**DOI:** 10.3390/ijms131013030

**Published:** 2012-10-11

**Authors:** Vasileios Askoxylakis, Volker Ehemann, Shoaib Rana, Susanne Krämer, Nuh N. Rahbari, Jürgen Debus, Uwe Haberkorn

**Affiliations:** 1 Department of Radiation Oncology, University of Heidelberg, INF 400, 69120, Heidelberg, Germany; E-Mails: shoaib.rana@dkfz.de (S.R.); juergen.debus@med.uni-heidelberg.de (J.D.); 2 Institute of Pathology, University of Heidelberg, INF 220, 69120, Heidelberg, Germany; E-Mail: volker.ehemann@med.uni-heidelberg.de; 3 Department of Nuclear Medicine, University of Heidelberg, INF 400, 69120, Heidelberg, Germany; E-Mails: susanne.kraemer@med.uni-heidelberg.de (S.K.); uwe.haberkorn@med.uni-heidelberg.de (U.H.); 4 Department of General, Visceral and Transplantation Surgery, University of Heidelberg, INF 110, 69120, Heidelberg, Germany; E-Mail: nuh.rahbari@med.uni-heidelberg.de

**Keywords:** peptide, phage display, colorectal carcinoma, targeting, imaging

## Abstract

Phage display represents an attractive screening strategy for the identification of novel, specific binding ligands that could be used for tumor targeting. Recently, a new peptide (CaIX-P1) with affinity for human carbonic anhydrase IX (CAIX) was identified and evaluated. The aim of the present study is to characterize the properties of CaIX-P1 for targeting human colorectal carcinoma and investigate the correlation of peptide binding with the expression of carbonic anhydrase IX. Human colorectal carcinoma HCT116 and HT29 cells were investigated for CAIX expression using Western Blot analysis. Binding and competition studies of ^125^I-radiolabeled CaIX-P1 were performed on HCT116 cells *in vitro*. FACS analysis and fluorescence microscopy studies were carried out after cell incubation with fluorescein-labeled CaIX-P1 and rhodamine-labeled anti-human CAIX-mAb. Our studies revealed an enhanced *in vitro* expression of carbonic anhydrase IX in HCT116 and HT29 cells with increasing cell density. Binding of ^125^I-labeled-CaIX-P1 on HCT116 cells increased with increasing cell density and correlated to the CAIX expression. FACS analysis demonstrated a correlation of cell labeling between FITC-CaIX-P1 and rhodamine-labeled anti-CAIX-mAb in both HCT116 and HT29 cells. The results of our study indicate that the phage display identified peptide CaIX-P1 might be an attractive candidate for the development of a ligand targeting CAIX in colorectal cancer.

## 1. Introduction

Carbonic anhydrase IX (CAIX) is a membrane-associated zinc metalloenzyme, known to be over-expressed in various human tumors, including renal, ovarian, lung and colorectal carcinomas [1]. The protein catalyzes the reversible hydration of carbon dioxide and has a key role in pH regulation, whereas it is involved in various cellular processes including cell proliferation and adhesion. At the molecular level, it has been demonstrated that *CAIX* is a target gene of the hypoxia inducible factor 1α (HIF-1α), a transcription factor that accumulates under conditions of low oxygen concentration and activates a series of genes, which are responsible for cellular response to hypoxia [2]. The expression of carbonic anhydrase IX in tumors is associated with a poor disease prognosis, most probably due to the correlation of the protein with low oxygen levels [3]. Decreased oxygenation in solid tumors causes molecular and phenotypic changes characterized by aggressive and rapid growth and poor responsiveness to therapeutic approaches, such as radio- or chemotherapy [4]. The fact that *CAIX* is considered to be an intrinsic marker of tumor hypoxia and that its’ expression is associated with an adverse disease prognosis make the protein an attractive candidate for the development of targeting strategies. In this respect, emphasis must be put on the potential of CAIX for non-invasive imaging purposes. *In vivo* visualization of CAIX expression in tumors could be used for the prediction of treatment response and outcome, which is of high importance, because it might provide a rationale for patient stratification during treatment decision making.

Within the past years different molecules with affinity for human carbonic anhydrase IX have been identified. Prominent examples are sulfonamides and monoclonal antibodies. Sulfonamides are known to have a good affinity for CAIX and represent attractive candidates for the development of therapeutic agents that target pH regulators in tumor cells [5]. In regard however to imaging applications, the targeting features of small molecules are often limited by a low specificity. In particular, many sulfonamides bind to carbonic anhydrase II (CAII), which is expressed in human erythrocytes [6], resulting in unfavorable increased background activity. In more recent years major progress has been done, leading to the development of new sulfonamides and coumarin compounds with increased CAIX selectivity [7]. Preclinical studies of such agents within imaging [8] and therapeutic [9] approaches revealed very encouraging results, still further pharmacologic work is necessary prior to their use in clinical approaches. Monoclonal antibodies or antibody fragments targeting CAIX on the contrary are characterized by very high target specificity [10]. However, they also possess unfavorable properties for imaging strategies, mainly due to their large size, which results in very slow blood clearance, high background noise for extended time periods and reduced imaging contrast [11]. Therefore, there is a need for new specific binding molecules with improved pharmacokinetic features, such as effective tumor penetration and rapid blood clearance. Attractive ligands in this respect are peptides [12].

Novel peptides with specific binding affinity for a target can be identified using high throughput screening techniques. A prominent example is phage display. The technology offers a wide spectrum of applications, including (i) identification of new receptors, natural ligands and high-affinity antibodies and analogues; (ii) mapping and mimicking of epitopes, and (iii) isolating specific antigens that bind to bioactive compounds. Screening large peptide libraries has led to the development of specific molecules binding tumor cells or the tumor vasculature [13].

Recently phage display was used for identification of the peptide CaIX-P1 (YNTNHVPLSPKY) targeting the extracellular domain of human carbonic anhydrase IX. This ligand was evaluated *in vitro* and *in vivo* for binding affinity, specificity, kinetics and cellular internalization, as well as for bio-distribution. The results indicated a specific affinity for human CAIX, allowing the conclusion that CaIX-P1 is an attractive lead structure for the development of molecules specifically targeting the protein [14].

To avoid bias due to varying CAIX expression, the new peptide ligand CaIX-P1 was investigated in the initial study on renal cell carcinoma SKRC52 cells, stably over-expressing the target protein. However, the expression of CAIX in solid tumors is usually not stable but varies, dependent on microenvironment conditions within the inhomogeneous tumor tissue. Therefore, an issue that needs to be addressed is whether the peptide CaIX-P1 can bind the target even under conditions of differential CAIX expression. Aim of the present study was to investigate this aspect. We firstly evaluated the expression of human carbonic anhydrase IX in colorectal carcinoma HCT116 and HT29 cells under different conditions. The peptide CaIX-P1 was chemically synthesized, radiolabeled and tested for binding on HCT116 cells under conditions of variable CAIX expression. Furthermore, binding of CaIX-P1 was compared to cell binding of an anti-human carbonic anhydrase IX monoclonal antibody (anti-CAIX-mAb) using FACS analysis in both HCT116 and HT29 cells. To further characterize the affinity and specificity of radiolabeled CaIX-P1, competition experiments using the unlabeled peptide as competitor were carried out and the *in vivo* distribution of CaIX-P1 was evaluated in mice carrying subcutaneously transplanted colorectal carcinoma HCT116 tumors. Within our study human pancreatic carcinoma BxPC3 cells were used as negative control target. Our results reveal an *in vitro* correlation between CaIX-P1 binding and expression of human carbonic anhydrase IX in colorectal cancer cells, supporting the hypothesis that CaIX-P1 might be an attractive ligand for the development of CAIX affine molecules.

## 2. Results and Discussion

### 2.1. CAIX Expression and Biding of CaIX-P1

#### 2.1.1. CAIX Expression in HCT116, HT29 and BxPC3 Cells

**Figure 1:**
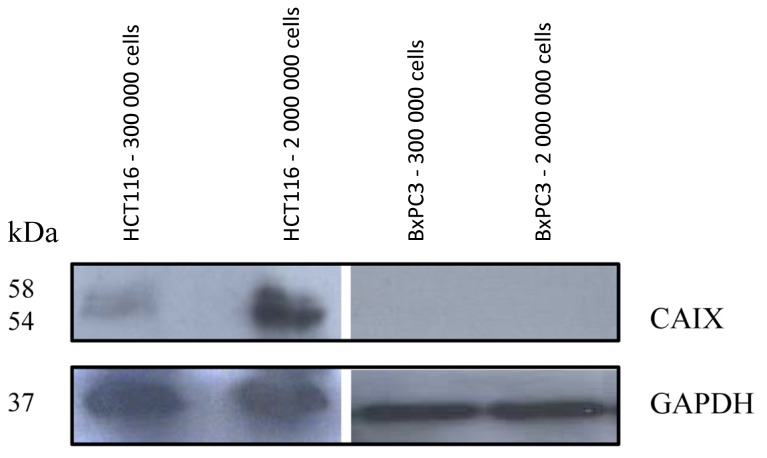
Western Blot analysis of HCT116 and BxPC3 cells at two different cellular densities. GAPDH was used as loading control.

Previous investigations of *CAIX* mRNA regulation in colorectal carcinoma HCT116 cells using real time PCR indicated a cell density dependent expression [14]. To prove the hypothesis that the protein is over-expressed under conditions of increased cell density, HCT116 cells were incubated to different levels of confluence and Western Blot analysis was performed. The Western Blot analysis revealed an up-regulation of CAIX at increased cell density as expected (Figure 1). Using the human pancreatic carcinoma cell line BxPC3 as negative control, no CAIX expression was noticed at increased cell density (Figure 1).

**Figure 2:**
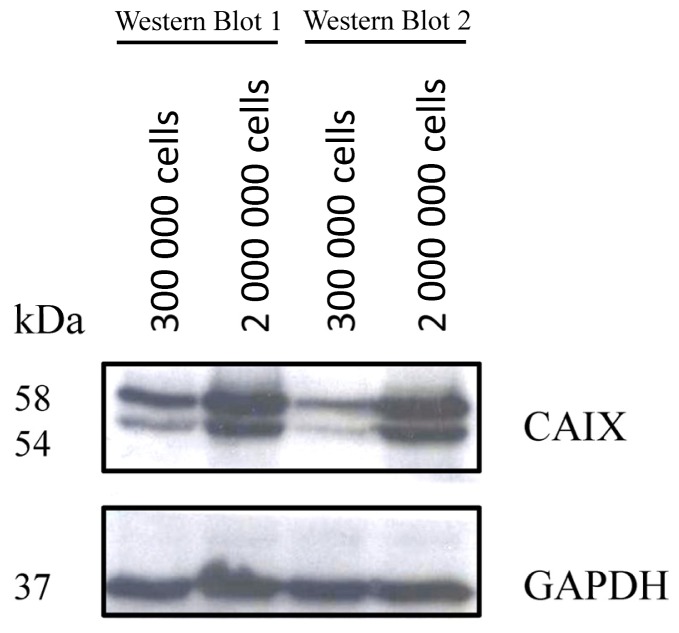
Western Blot analysis of HT29 cells at two different cellular densities. GAPDH was used as loading control. The experiment was performed in duplicate.

To further investigate this effect, the same experiments were applied on the colorectal adenocarcinoma cell line HT29. These experiments also demonstrated a density dependent up-regulation of human CAIX (Figure 2).

**Figure 3:**
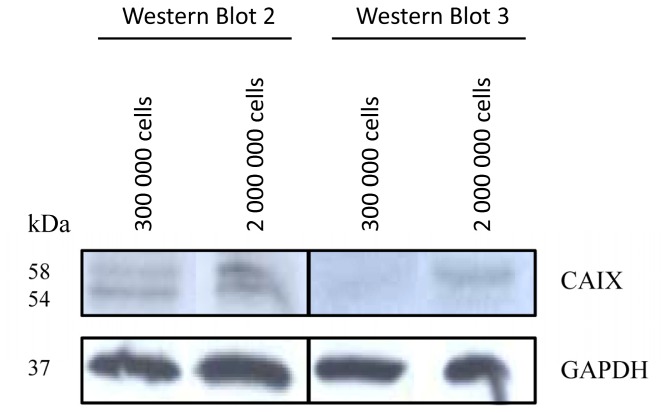
Western Blot analysis of different cultures of HCT116 cells treated under the same conditions of cellular density. GAPDH was used as loading control.

The density dependent up-regulation of CAIX in colorectal carcinoma HCT116 and HT29 cells is in concert with previous studies. In particular, Sansone *et al.* showed an increased CAIX expression at high cell density for colorectal carcinoma cells [15]. The density dependent CAIX up-regulation has furthermore been demonstrated for other tumor entities, such as breast and ovarial carcinoma [16]. A possible explanation is that culture conditions of high cell density create a pericellular hypoxic environment, which promotes the accumulation of the hypoxia inducible factor 1 and subsequently the expression of its target genes, including *CAIX* [15]. Since however the pericellular microenvironment cannot be completely controlled, an issue that might be raised is the exact reproducibility of the CAIX expression at conditions of differential cell density. This has been investigated through Western Blot analysis of CAIX expression in different cultures of HCT116 cells. Although the results of this analysis revealed that the absolute CAIX expression under the same conditions of cellular density may differ from culture to culture, they also demonstrated that in all cases a relative protein over-expression was induced under enhanced cellular density (Figure 3).

Alternative assays for establishment of differential protein expression include in vitro studies under conditions of stable hypoxia using either an established hypoxia chamber or enzymatic assays [17]. Studies under controlled and stable conditions of tumor hypoxia are very important, since hypoxia is a major factor of treatment resistance with high pathophysiological significance. However, in regard to carbonic anhydrase IX previous studies provided evidence for alternative pathways controlling the protein expression. In particular, Kaluz *et al.* demonstrated that although such pathways are also O_2_ sensitive, they differ in the threshold required for their initiation [18]. The authors showed that increased cellular density generates intermediate O_2_ levels, which do not increase the amount of HIF-1a but activate PI3K, affecting the induction of CAIX expression. Furthermore, they conclude that this model is consistent with what is seen in human tumors, where CAIX is not only expressed in hypoxic regions but also in areas that are not hypoxic [18]. This conclusion, in combination with our results as well as results from other studies clearly demonstrating a CAIX up-regulation under increased cellular density, reveal that the model of differential CAIX expression applied in our study is still of high biological relevance and significance.

#### 2.1.2. Kinetics of ^125^I-CaIX-P1 in HCT116 and BxPC3 Cells

**Figure 4:**
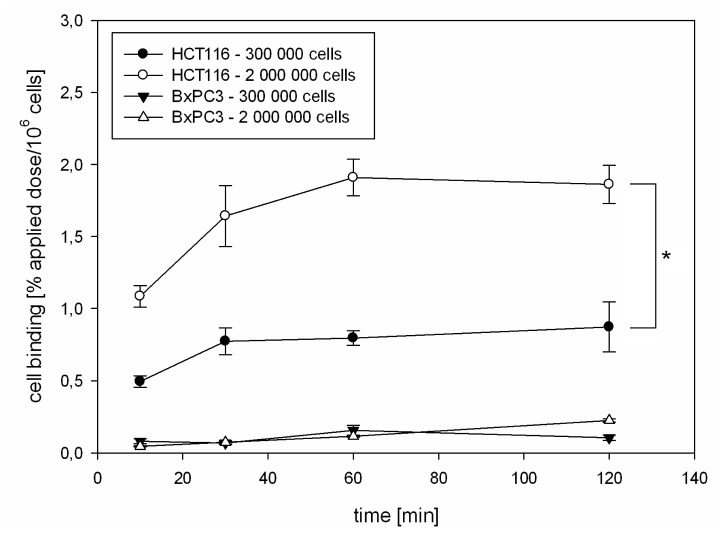
*In vitro* kinetics of ^125^I-CaIX-P1 on HCT116 and BxPC3 cells at two different cell densities. Mean values and standard deviation.

After establishing conditions of differential CAIX regulation, binding of the ^125^I-labeled CaIX-P1 peptide (YNTNHVPLSPKY) was investigated on HCT116 and BxPC3 cells. Kinetics studies were performed at conditions of two different cellular densities. To avoid bias through the different cellular confluence, normalization for the number of vital cells was performed and the radioligand uptake was calculated as percent applied dose per 10^6^ cells. These experiments demonstrated a significantly increased binding of ^125^I-CaIX-P1 per 10^6^ HCT116 cells with increasing cell density (* *p* < 0.05), revealing a correlation with the target protein expression as investigated by Western Blot analysis (Figure 4). The same experiments on BxPC3 cells at different cell densities revealed a strongly decreased binding to the background level. This result also correlated with the Western Blot analysis showing no CAIX expression in BxPC3 cells (Figure 1). Although our studies indicate a correlation between CAIX expression and peptide binding, the fact that low oxygen levels represent well established conditions for CAIX up-regulation, as well as that hypoxia is of high pathophysiological significance in tumor biology reveal the necessity for further detailed investigation of the binding properties of CaIX-P1 under controlled conditions of stably decreased oxygen concentrations.

#### 2.1.3. Fluorescence Microscopy Experiments

**Figure 5:**
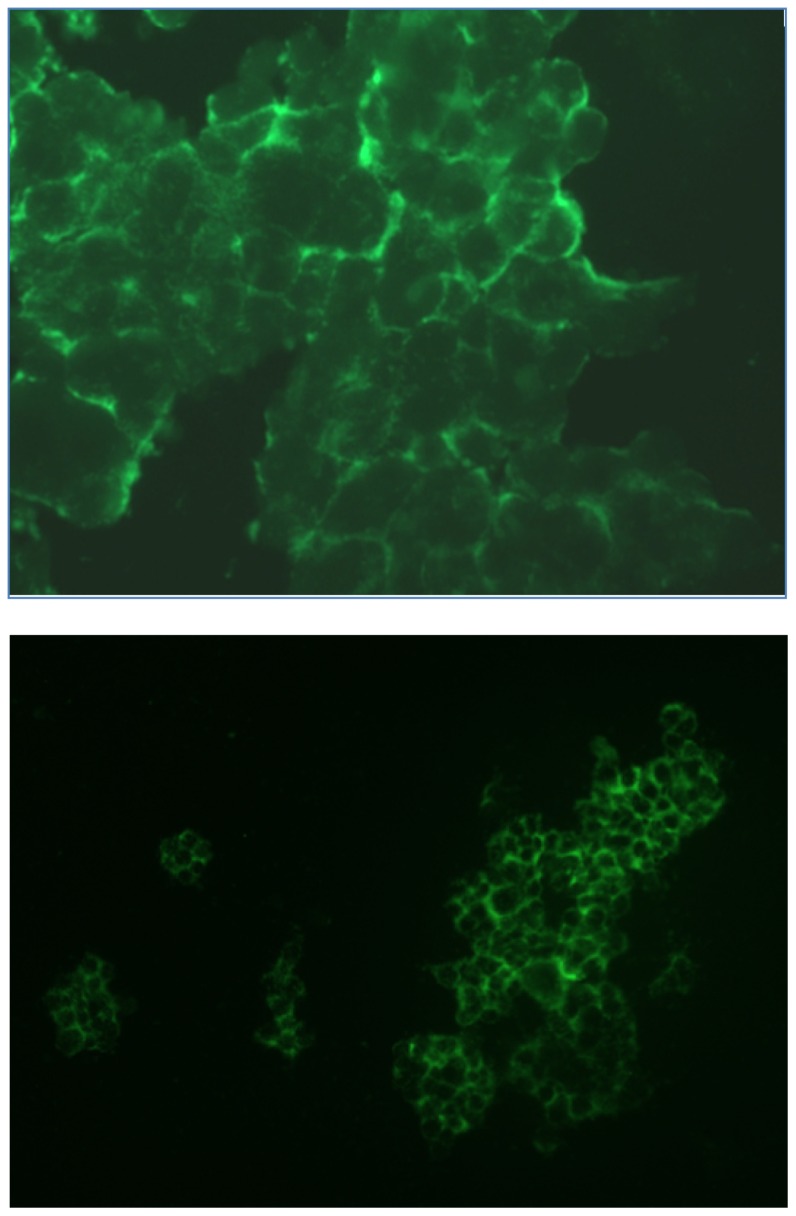
Fluorescence microscopy of FITC-labeled CaIX-P1. Images were performed from two different regions after incubation of HCT116 cells with FITC-CaIX-P1.

For fluorescence microscopy experiments FITC was coupled at the *N*-terminus of the amino acid sequence of CaIX-P1. The FITC-labeled peptide was incubated with HCT116 cells, which were allowed to flatten down and grow to high density. Analysis was performed after cell fixation on a Leica fluorescence microscope. This analysis revealed an intensive fluorescence signal at the periphery of HCT116 cells in regions of high cellular density (Figure 5). This result is in concert with the previous data, showing an increased protein expression at high cellular density and a correlation with the radioligand binding.

**Figure 6:**
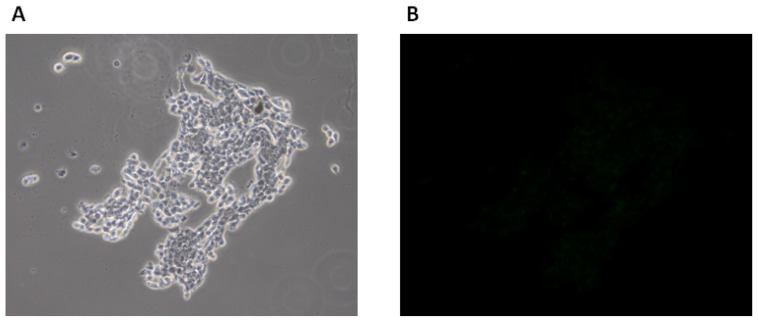

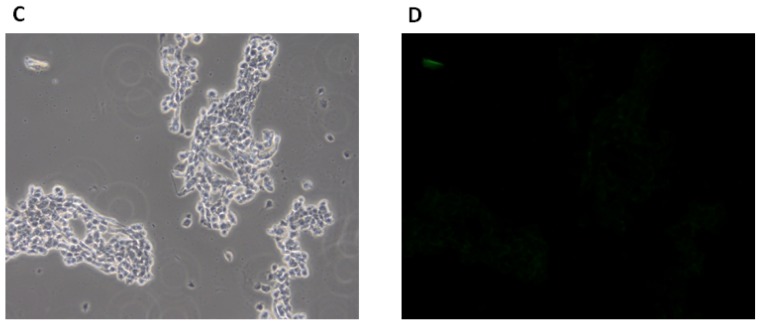
Fluorescence microscopy of HCT116 cells after treatment with fluorescein. (**A** and **C**): Phase contrast images of different regions. (**B** and **D**): Respective fluorescence microscopy images of the same regions.

As negative control HCT116 cells were incubated with fluorescein alone at the same concentration and under the same conditions. Fluorescence microscopy analysis of cells treated with fluorescein alone showed a strongly decreased signal to the background level, supporting the hypothesis that cell labeling is mediated by the FITC-labeled CaIX-P1 peptide and not by fluorescein (Figure 6).

### 2.2. Fluorescence-Activated Cell Sorting (FACS)

**Figure 7:**
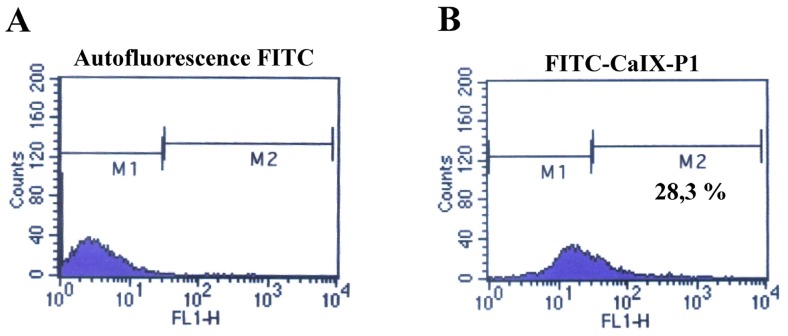

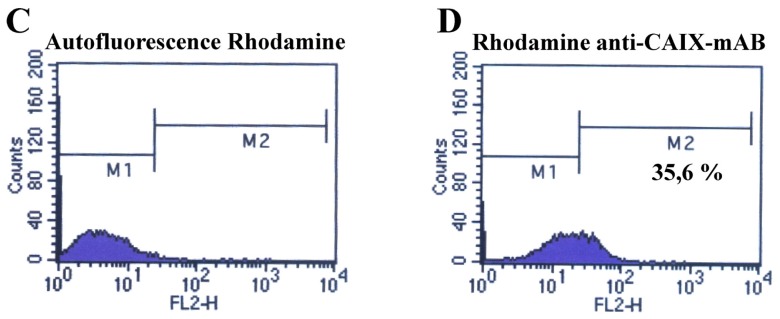
Fluorescence-activated cell sorting analysis. (**A**) Autofluorescence of HCT116 cells at 530nm (FL1). (**B**) Fluorescence of HCT116 cells after incubation with FITC-CaIX-P1. (**C**) Autofluorescence of HCT116 cells at 590nm (FL2). (**D**) Fluorescence of HCT116 cells after incubation with rhodamine-labeled anti-CAIX-mAb.

To further characterize the correlation between the up-regulation of carbonic anhydrase IX at increased cell density and the binding of the peptide CaIX-P1, fluorescence-activated cell sorting (FACS) studies were carried out. For FACS analysis HCT116, HT29 and BxPC3 cells were grown to high cellular density and incubated at the same conditions either with a rhodamine- or Alexa fluor-labeled anti-human carbonic anhydrase monoclonal antibody (anti-CAIX-mAb) or with FITC-labeled CaIX-P1 or with both. The FACS analysis demonstrated similar portions of labeled cells for the antibody and the CaIX-P1 peptide when the cells were cultivated under the same conditions. In particular, after determination of the cell autofluorescence, about 36% and 28% of HCT116 cells were found to be labeled with the anti-CAIX mAb and the CaXI-P1 peptide respectively (Figure 7).

**Figure 8:**
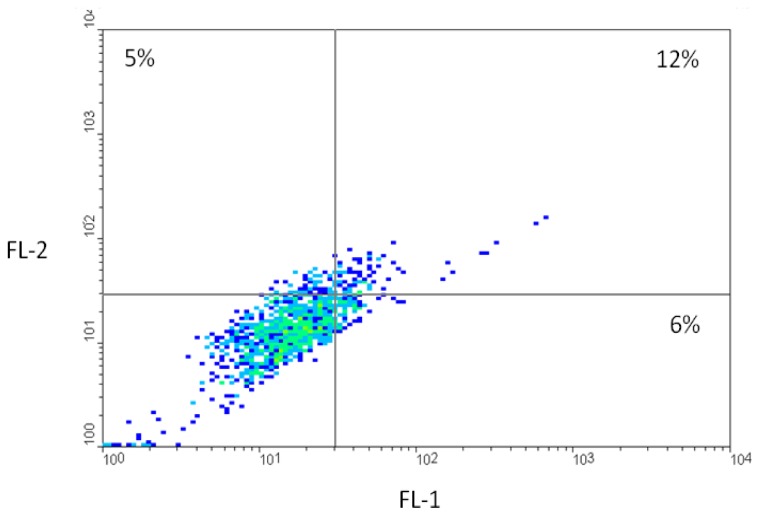
Fluorescence-activated cell sorting analysis after co-icubation of HCT116 cells with FITC-labeled CaIX-P1 and rhodamine-labeled anti-human CAIX mAb. **Low left box**: unlabeled cells (autofluorescence). **Low right box**: cells labeled only with FITC-CaIX-P1. **Upper left box**: cells labeled only with rhodamine-anti-CAIX-mAb. **Upper right box**: Cells labeled with both FITC-CaIX-P1 and rhodamine-anti-CAIX-mAb.

Co-incubation of HCT116 cells at the same conditions with both molecules (rhodamine-labeled anti-human CAIX mAb and FITC-labeled CaIX-P1) revealed that about 71% of the cells labeled with anti-human CAIX mAb were co-labeled with FITC-CaIX-P1 (Figure 8). In particular, after determination of autofluorescence at 530 nm (FL-1) and 590 nm (FL-2) (Cutoff lines Figure 8), 17% of all cells were found to be labeled with rhodamine-anti-CAIX-mAb (upper boxes Figure 8). Among them only 5% was within the autofluorescence level at 530 nm (upper left box, Figure 8). The rest (12% of all cells, upper right box, Figure 8) showed a signal, which was higher than the autofluorescence signal at 530 nm, indicating that about 71% (12%/17%) of the cells labeled with anti-human CAIX mAb were co-labeled with FITC-CaIX-P1.

**Figure 9:**
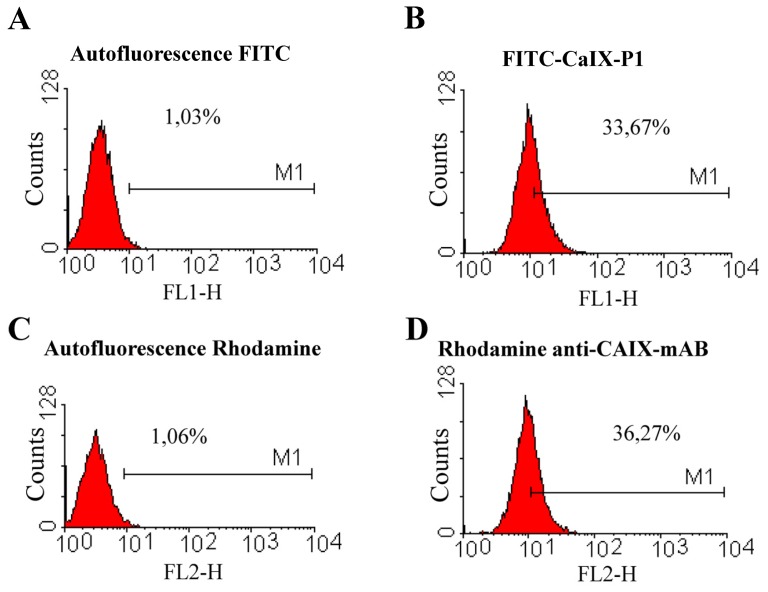
Fluorescence-activated cell sorting analysis. (**A**) Autofluorescence of HT29 cells at 530nm (FL1). (**B**) Fluorescence of HT29 cells after incubation with FITC-CaIX-P1. (**C**) Autofluorescence of HT29 cells at 590nm (FL2). (**D**) Fluorescence of HT29 cells after incubation with rhodamine-labeled anti-CAIX-mAb.

In addition to HCT116 cells, FACS analysis of HT29 cells incubated with FITC-CaIX-P1 or rhodamine labeled anti-CAIX mAb under conditions of increased cellular density revealed that about 33% of all cells were labeled FITC-CaIX-P1, whereas about 36% of all cells were labeled with rhodamine-anti-CAIX mAb (Figure 9).

**Figure 10:**
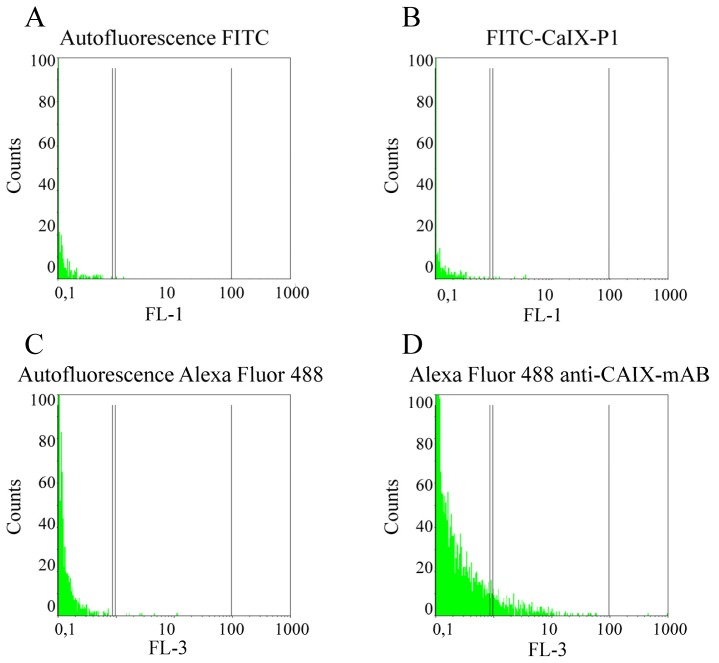
Fluorescence-activated cell sorting analysis. (**A**) Autofluorescence of BxPC3 cells at 530nm (FL1). (**B**) Fluorescence of BxPC3 cells after incubation with FITC-CaIX-P1. (**C**) Autofluorescence of BxPC3 cells at 488nm (FL3). (**D**) Fluorescence of BxPC3 cells after incubation with Alexa Fluor-labeled anti-CAIX-mAb.

The same experiments using the CAIX negative human pancreatic carcinoma cell line BxPC3 revealed only background level binding of both Alexa fluor-labeled anti-human CAIX mAb and FITC labeled CaIX-P1 (Figure 10).

These results indicate that the novel peptide CaIX-P1 possibly recognizes the extracellular domain of carbonic anhydrase IX in a similar manner like the monoclonal antibody and strengthens therefore the hypothesis that the dodecapeptide might be used as a lead structure for the development of specific molecules targeting CAIX in tumors.

### 2.3. Competition Studies on HCT116 Cells

**Figure 11:**
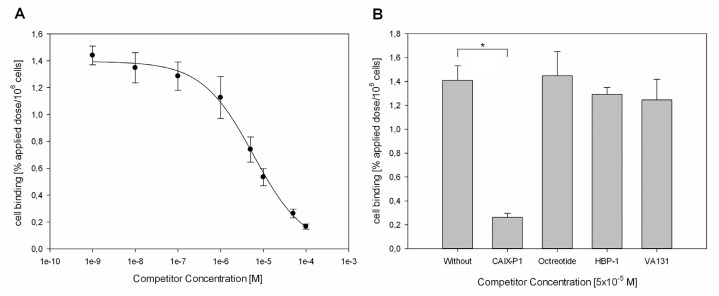
(**A**) Binding inhibition of ^125^I-CaIX-P1 by the unlabeled CaIX-P1 peptide at various concentrations on HCT116 cells. (**B**) Specific binding of ^125^I-CaIX-P1 on HCT116 cells. Non-specific binding was determined through co-incubation with 5 × 10^−5^ M unlabeled CaIX-P1. Octreotide, HBP-1 and VA131 were used as negative control competitors at the same concentration.

To evaluate the binding specificity of CaIX-P1 on colorectal carcinoma HCT116 cells competition experiments were performed as previously described [14]. Co-incubation of ^125^I-CaIX-P1 with the unlabeled peptide at different concentrations revealed an increasing inhibition of radioligand binding in the presence of the competitor (Figure 11A). Maximal radioligand inhibition of about 90% was reached at a competitor concentration of 10^−4^ M (* *p* < 0.05). Using different ligands, such as octreotide and the peptides HBP-1 and VA131, as negative control competitors at the same concentration (5 × 10^− 5^ M), binding of the radioligand could not be competitively abolished (Figure 11B).

The negative control peptides HBP-1 and VA131 were randomly chosen. HBP-1 (SPRGDLAVLGHKY) is a linear peptide identified by phage display technology on squamous cell carcinoma cells of the head and neck [19], whereas VA131 (PWMEY) is a derivative of the phage display identified dodecapeptide p160 [20]. The fact that binding of the radioligand was strongly inhibited by the unlabeled CaIX-P1 peptide in a concentration dependent manner but was not affected by the negative control linear peptides supports the hypothesis of a specific target binding. Similar results were previously reported for the stably CAIX expressing cell line SKRC52 [14]. However, although our results strengthen the hypothesis of a specific ligand binding, they also reveal an IC^50^ value of about 1–5 μM, which is low compared to peptide-based molecules that are used in clinical targeting approaches. Prominent examples are the somatostatin analogon octreotide and the αvβ3 integrin targeting RGD sequence, which show nanomolar binding affinities towards their targets [21,22]. Therefore, a major issue of further investigation is the enhancement of the ligand’s affinity. An essential step in this direction is the identification of the amino acids in the sequence of CaIX-P1 that are important for target binding. Identification of key amino-acid residues can be achieved by various strategies, including peptide truncation from the *N*- or *C*-termini for isolation of the minimum binding sequence, or amino-acid scan, such as alanine-scan, for characterization of the relative importance of particular functional groups in the peptide sequence [23]. Recent studies applying such strategies revealed modifications in the sequence of the peptide, which might be favorable for improvement of the ligand’s target affinity and indicated a fragment (CaIX-P1-4-10) with increased binding capacity [24]. However, our study also demonstrated that such approaches require the synthesis and moreover the evaluation of a high number of derivatives, which is time and cost intensive. An attractive alternative method is the use of peptide arrays [25]. This technology is based on the principle that hundreds upon thousands of peptides are synthesized and presented on a planar surface at a time [26]. Incubation of the target with the peptide array and analysis of interactions between target and the spotted sequences can lead to identification of molecules with increasing binding affinity. In recent studies, peptide arrays were successfully applied for the identification of peptide-cell interactions [27].

Once the essential amino acids for target binding are determined, establishment of the optimal ligand conformation is required for affinity optimization. Linear peptides are characterized by increased 3D flexibility and enhanced loss of conformational entropy upon formation of the ligand-target-complex. Local or global conformational constraints can stabilize the structure, which is optimally complementary to the binding site. Targeted modifications to constrain the local conformation include for example the use of D-amino acid residues, which can stabilize a reverse β-turn structure [23]. Global conformational constraints such as cyclization of the peptidyl backbone or grafting of the binding domain into a molecular scaffold, potentially lead to an increased structural rigidity and a higher Gibbs free binding energy [28], facilitating an affinity increase.

### 2.4. *In Vivo* Bio-Distribution

**Figure 12:**
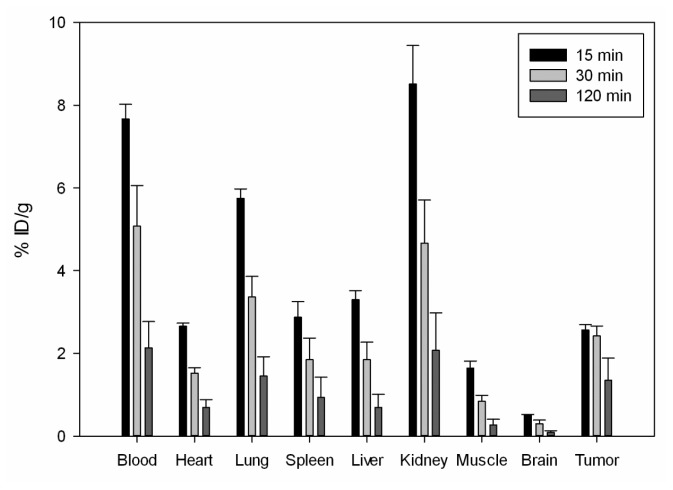
Organ distribution experiments of ^131^I-labeled CaIX-P1 in Balb/c nu/nu mice, carrying HCT116 tumors (3 animals per experiment).

A prerequisite for the *in vivo* use of an agent as targeting vehicle is a selective binding to the tissue of interest. This requirement is fulfilled when the uptake by the healthy tissues is lower than that of the target in bio-distribution experiments. The organ distribution data of ^131^I-labeled CaIX-P1 in mice bearing HCT116 tumors indicates that the peptide has potential in this respect, however demonstrates at the same time major challenges prior to a possible *in vivo* use of the molecule. In particular, the results of the organ distribution experiments reveal a higher uptake in the tumor after 30 min and 120 min circulation, compared to most healthy tissues, such as heart, spleen, liver, muscle and brain (Figure 12).

Furthermore, our studies showed that although tumor binding of CaIX-P1 decreased with time progression, a higher decrease was noticed for the healthy tissues resulting in a significant increase of the tumor-to-organ ratios over time (* *p* < 0.05) (Table 1).

The biodistribution experiments also indicated a slight correlation between the tumor size and radioligand uptake. In particular both at 30 min and 120 min p.i. lower tumor uptake (%ID/g tissue) and lower tumor-to-organ ratios were noticed for animals carrying subcutaneously tumors with a weight of ≤100 mg (data not shown) compared to animals with larger tumors. This result seems to be in concert with the *in vitro* data showing a correlation between cell density and radioligand uptake, it must be however, considered very critically and proved in further experiments, including immunohistochemistry and autoradiography studies, in detail. Main reason for that is the metabolic instability of the peptide. Previous investigation of serum stability revealed a rapid peptide degradation by serum proteases. The half-life was measured to be about 25 min [14]. Therefore, a prerequisite for safe conclusions on the *in vivo* uptake properties of CaIX-P1 is the improvement of the peptide’s metabolic characteristics.

A further important issue that is associated with the proteolytic degradation of CaIX-P1 is the elevated levels of radioactivity in blood. Elevated blood values represent a major drawback, since they cause increased background noise that is unfavorable for imaging applications. Metabolic instability of CaIX-P1 is found to be associated with labeling instability, which further increases the blood background. It has been shown in previous studies that the product of serum degradation is a derivative lacking the *N*-terminal tyrosine [14]. Since radioiodination is performed on the tyrosine residues, free radiolabeled tyrosine molecules might be produced, which then circulate in the blood stream and are responsible for increased background noise. Therefore, further studies must focus on two major aspects: the first aspect includes the improvement of the metabolic stability. This can be achieved by modifications in the amino-acid sequence, including use of unnatural amino-acids which cannot be recognized by proteases, such as D-isoforms, *N*-terminal acetylation or grafting of the binding sequence in a scaffold structure [29,30]. However, such modifications might induce conformational changes to the specific binding molecule and further reduce its binding affinity [31]. Furthermore, although scaffolds are successfully applied to improve the serum stability, they also lead at the same time to a significant increase of the ligand’s molecular size influencing the advantageous pharmacokinetic properties of oligopeptides, such as improved tumor penetration and rapid clearance. The second aspect refers to the improvement of radiolabeling stability. Alternatively to direct iodination, indirect labeling via a chelating moiety, covalently bound to the peptide, can be performed [32]. Macrocyclic chelators, such as DOTA or branched chelators, such as DTPA, which utilize carboxylate and amine groups to form stable complexes with radioactive metals, provide improved labeling stability [33], have however also the disadvantage that they might change the conformational structure of the specific binding ligand and negatively influence its target affinity.

## 3. Experimental Section

### 3.1. Cell Lines

Human colorectal carcinoma HCT116 cells and human pancreatic carcinoma BxPC3 cells were cultivated in RPMI 1640 cell culture medium supplemented with 10% (*v*/*v*) fetal calf serum (Invitrogen). Human colorectal adenocarcinoma HT29 cells were cultivated in DMEM supplemented with 10% FCS.

### 3.2. Peptides

The peptides CaIX-P1 (YNTNHVPLSPKY), HBP-1 (SPRGDLAVLGHKY) and VA131 (PWMEY) were chemically synthesized on an ABI 433 A peptide synthesizer (Applied Biosystems) as described previously [14]. After HPLC based purification (Chromolith Semi Prep Column RPe18, 10 × 100 mm, Merck, Darmstadt, Germany), lyophilization and mass spectrometry analysis (MALDI-3; Kratos instruments), the peptide was radiolabeled with ^125^I or ^131^I using the chloramine T method and the purified product was analyzed on a Chromolith Performance RP-18e 10 × 4.6 mm column (Merck). All experiments were conducted under the same radiolabeling conditions. For fluorescence microscopy studies and fluorescence-activated cell sorting, fluorescein isothiocyanate (FITC) was coupled at the *N*-terminus of CaIX-P1.

### 3.3. *In Vitro* Binding Studies

300,000 and 2,000,000 human colorectal carcinoma HCT116 and pancreatic carcinoma BxPC3 cells were cultivated in 6-well plates for 48 h. RPMI 1640 medium with 1% BSA was used for cell blocking. Thereafter, 1 mL RPMI 1640 medium containing about 1.0 × 10^6^ cpm ^125^I-labeled peptide (specific activity ~50 GBq/μmol) was added and cell incubation was carried out for various time periods (10–120 min). To characterize the specificity of radioligand binding, co-incubation with unlabeled CaIX-P1 at various concentrations (10^−4^ to 10^−9^ mol/L) was performed. After incubation the cells were washed with ice cold PBS and lysed with 0.5 mL NaOH 0.3 mol/L. The lysate was collected and the radioactivity was measured with a γ-counter (LB951G; Berthold Technologies). Bound radioactivity, representing the amount of peptide accumulation on HCT116 cells, was calculated as percentage applied dose per 10^6^ cells. Binding specificity was further determined using octreotide, HBP-1 and VA131 as negative control competitors at the same concentration as CaIX-P1. All binding and competition experiments were performed in serum free medium in order to avoid peptide degradation by serum proteases.

### 3.4. Western-Blot Analysis

300,000 and 2,000,000 HCT116, HT29 and BxPC3 cells were cultivated in 6-well plates at 37 °C for 48 h. After cell washing with 10 mL ice-cold PBS pH 7.4 and scraping with a cell scraper, the lysate was centrifuged for 3 min at 1000 rpm. The pellet was washed with 5 mL PBS and subsequently centrifuged for 3 min at 1000 rpm. 2 mL Triton X-100 1% was added to the pellet and centrifugation was performed for 10 min at 2700 rpm. After protein concentration determination, equal amounts of protein were loaded into a polyacrylamide gel, separated by electrophoresis and transferred to a nitrocellulose membrane with a Mini Trans-Blotter (100 V for 90 min). Membranes were blocked with 5% non-fat milk powder in TBST buffer for 1 h at room temperature. Incubation overnight with a primary rabbit IgG monoclonal anti-human CAIX antibody (Abcam, ab108351; 1:1000 dilution) was performed at 4 °C. Thereafter the membrane was washed with TBST and incubated with horseradish peroxidase conjugated antibody (R & D Systems, HAF008; 1:1000 dilution) at room temperature for 60 min. As control, anti-GAPDH antibody (Abcam; 1:5000 dilution) and polyclonal anti-mouse (DakoCytomation, P 0447; 1:1000 dilution) were respectively used. Antibody binding was determined using a chemoluminescence detection system.

### 3.5. Fluorescence Microscopy Experiments

Human colorectal carcinoma HCT116 cells were seeded in culture plates (1,000,000–2,000,000 cells/well in 3 mL medium) containing glass coverslips and were allowed to grow for 48 h. The medium was replaced by 900 μL fresh medium (without FCS) and 100 μL 10^−4^–10^−5^ M sterilized stock solution of FITC-labeled-CaIX-P1 or fluorescein at the same concentration was added to the cells. The plates were incubated for 1 h at 37 °C. After incubation the cells were washed thrice with 1 mL ice cold PBS (pH 7.4). Subsequently the cells were fixed with 2% formaldehyde in PBS (pH 7.4) at room temperature for 20 min. This procedure was performed on ice. The cells were washed with ice cold PBS (1 × 600 μL, pH 7.4) and the cover slips were mounted on a slice with DAKO fluorescent medium. Cells were photographed using a Leica DM-RA2 fluorescence microscope. Digitalized figures were processed using standard imaging software (Adobe Photoshop 6.0/Illustrator 9.0).

### 3.6. Fluorescence-Activated Cell Sorting (FACS)

For fluorescence activated cell sorting 1,000,000–2,000,000 HCT116, HT29 or BxPC3 cells were seeded into 6-well plates and cultivated in 3 mL incubation medium at 37 °C for 48 h. The medium was replaced by 1 mL fresh medium (without FCS) containing FITC-CaIX-P1 at a concentration of 10^−5^–10^−6^ mol/L or 5–10 μg mouse anti-human CAIX-mAb (R & D Systems, MAB2188) or both. After overnight incubation the medium was removed and the cells were washed with 3 mL PBS. Cells incubated with anti-human CAIX-mAb were further incubated with a rhodamine conjugated anti-mouse IgG (Rockland, 610-4002) or an Alexa fluor conjugated IgG overnight. The cells were transferred in 1.5 mL Eppendorf tubes and centrifuged at 1000 rpm for 10 min. The pellet was resuspended in cell wash medium and fluorescence activated cell sorting was performed. To discriminate between labeled cells and autofluorescence of unlabeled cells, autofluorescence of HCT116, HT29 or BxPC3 cells was measured and determined through a cutoff line. Cells that showed a higher fluorescence signal than the cutoff value were considered labeled by FITC-CaIX-P1 or rhodamine/Alexa fluor labeled anti-CaIX-mAb. Flow cytometry analysis was performed in a FACS-Calibur (Becton & Dickinson) equipped with a 488 nm argon laser with filter combination for FITC (530 nm) and rhodamine (590 nm). Histogram and dotplot analysis were performed using the Cellquest software (Becton & Dickinson).

### 3.7. *In Vivo* Organ Distribution

*In vivo* experiments were performed in Balb/c nu/nu mice, obtained from Charles River WIGA. Human colorectal carcinoma HCT116 tumors were transplanted through subcutaneous injection of a cell suspension (~5 × 10^6^ cells in OPTI-MEM) into the mouse trunk. After the tumors were grown to a size of 1.0 cm^3^, approximately 1 MBq of ^131^I-labeled CaIX-P1 was injected into the tail vein. The radioligand circulated in the mice for 15, 30 and 120 min and thereafter the animals were euthanized. Tumor, blood and selected organ tissues including heart, lung, spleen, liver, kidney, muscle and brain were removed and drained of blood. The organs were weighed and radioactivity was measured in a γ-counter. The tumor and organ uptake was calculated as percentage injected dose per gram tissue (%ID/g). All animal experiments were carried out in conformity with German and European laws for animal protection. The animal studies were approved by the Regierungspräsidium Karlsruhe, Baden-Württemberg, Germany, file reference: 35-9185.81/G-132/04.

### 3.8. Data Analysis and Statistics

Statistical analysis was performed using the unpaired Student t-test on the SIGMASTAT program (Jandel Scientific). A *p*-value of 0.05 or less was considered statistically significant.

## 4. Conclusions

In order to evaluate the potential of the novel peptide CaIX-P1 for targeting human carbonic anhydrase IX we investigated its binding characteristics under conditions of differential CAIX expression in HCT116 and HT29 human colorectal carcinoma cells. CAIX negative human pancreatic carcinoma BxPC3 cells were used as negative control target. We focused mainly on two major aspects: correlation of the peptide’s cellular binding with the expression of carbonic anhydrase IX and comparison to the binding of a monoclonal anti-human CAIX antibody under the same conditions of target expression. The results of our studies revealed an up-regulation of CAIX in colorectal carcinoma cells with increasing cell density. Both radiolabeled and fluorescence-labeled CaIX-P1 showed a significantly enhanced accumulation at high cell density levels. FACS analysis demonstrated similar rates of labeled cells with CaIX-P1 and anti-CAIX-mAb, whereas about 71% of the anti-CAIX-mAb labeled HCT116 cells were found to be co-labeled with the peptide. These results indicate that CaIX-P1 might be a promising lead structure for the development of oligopeptide molecules targeting carbonic anhydrase IX in tumors. However, further intensive studies are necessary in order to stabilize the ligand, improve its target affinity and bio-distribution and use it for *in vivo* applications.

## Figures and Tables

**Table 1:** Tumor-to-organ ratios calculated from the organ distribution studies.

Tumor to organ ratio	10 min *	30 min*	120 min *
Blood	0.335	0.482	0.622
Heart	0.965	1.593	1.914
Lung	0.447	0.723	0.915
Spleen	0.904	1.348	1.495
Liver	0.781	1.332	1.964
Kidney	0.304	0.526	0.656
Muscle	1.572	2.902	5.180
Brain	4.889	8.423	15.140

* *p* < 0.05.

## References

[1] Winum J.Y., Rami M., Scozzafava A., Montero J.L., Supuran C. (2008). Carbonic anhydrase IX: A new druggable target for the design of antitumor agents. Med. Res. Rev.

[2] Grabmaier K., A de Weijert M.C., Verhaegh G.W., Schalken J.A., Oosterwijk E. (2004). Strict regulation of CAIX(G250/MN) by HIF-1alpha in clear cell renal cell carcinoma. Oncogene.

[3] Pinheiro C., Sousa B., Albergaria A., Paredes J., Dufloth R., Vieira D., Schmitt F., Baltazar F. (2011). GLUT1 and CAIX expression profiles in breast cancer correlate with adverse prognostic factors and MCT1 overexpression. Histol. Histopathol.

[4] Vaupel P., Mayer A. (2007). Hypoxia in cancer: Significance and impact on clinical outcome. Cancer Metastasis Rev.

[5] Neri D., Supuran C.T. (2011). Interfering with pH regulation in tumours as a therapeutic strategy. Nat. Rev. Drug Discov.

[6] Supuran C.T., Winum J.Y., Wang B. (2009). Drug Design of Zinc-Enzyme Inhibitors–Functional, Structural, and Disease Applications.

[7] Supuran C.T. (2012). Inhibition of carbonic anhydrase IX as a novel anticancer mechanism. World J. Clin. Oncol.

[8] Dubois L., Lieuwes N.G., Maresca A., Thiry A., Supuran C.T., Scozzafava A., Wouters B.G., Lambin P. (2009). Imaging of CA IX with fluorescent labelled sulfonamides distinguishes hypoxic and (re)-oxygenated cells in a xenograft tumour model. Radiother. Oncol.

[9] Ahlskog J.K., Dumelin C.E., Trüssel S., Mårlind J., Neri D. (2009). *In vivo* targeting of tumor-associated carbonic anhydrases using acetazolamide derivatives. Bioorg. Med. Chem. Lett.

[10] Li J., Shi L., Wang C., Zhang X., Jia L., Li X., Zhou W., Qi Y., Zhang L. (2011). Preliminary biological evaluation of 125I-labeled anti-carbonic anhydrase IX monoclonal antibody in the mice bearing HAT-29 tumors. Nucl. Med. Commun.

[11] Wu A.M., Senter P.D. (2005). Arming antibodies: Prospects and challenges for immunoconjugates. Nat. Biotechnol.

[12] Haberkorn U., Eisenhut M., Altmann A., Mier W. (2008). Endoradiotherapy with peptides-status and future development. Curr. Med. Chem.

[13] Marr A., Markert A., Altmann A., Askoxylakis V., Haberkorn U. (2011). Biotechnology techniques for the development of new tumor specific peptides. Methods.

[14] Askoxylakis V., Garcia-Boy R., Rana S., Krämer S., Hebling U., Mier W., Altmann A., Markert A., Debus J., Haberkorn U. (2010). A new peptide ligand for targeting human carbonic anhydrase IX, identified through the phage display technology. PLoS One.

[15] Sansone P., Piazzi G., Paterini P., Strillacci A., Ceccarelli C., Minni F., Biasco G., Chieco P., Bonafé M. (2009). Cyclooxygenase-2/carbonic anhydrase-IX up-regulation promotes invasive potential and hypoxia survival in colorectal cancer cells. J. Cell. Mol. Med.

[16] Hunakova L., Bodo J., Chovancova J., Sulikova G., Pastorekova S., Sedlak J. (2007). Expression of new prognostic markers, peripheral-type benzodiazepine receptor and carbonic anhydrase IX, in human breast and ovarian carcinoma cell lines. Neoplasma.

[17] Askoxylakis V., Millonig G., Wirkner U., Schwager C., Rana S., Altmann A., Haberkorn U., Debus J., Mueller S., Huber P.E. (2011). Investigation of tumor hypoxia using a two-enzyme system for *in vitro* generation of oxygen deficiency. Radiat. Oncol.

[18] Kaluz S., Kaluzová M., Chrastina A., Olive P.L., Pastoreková S., Pastorek J., Lerman M.I., Stanbridge E.J. (2002). Lowered oxygen tension induces expression of the hypoxia marker MN/carbonic anhydrase IX in the absence of hypoxia-inducible factor 1 alpha stabilization: A role for phosphatidylinositol 3′-kinase. Cancer Res.

[19] Nothelfer E.M., Zitzmann-Kolbe S., Garcia-Boy R., Krämer S., Herold-Mende C., Altmann A., Eisenhut M., Mier W., Haberkorn U. (2009). Identification and characterization of a peptide with affinity to head and neck cancer. J. Nucl. Med.

[20] Zhang J., Spring H., Schwab M. (2001). Neuroblastoma tumor cell-binding peptides identified through random peptide phage display. Cancer Lett.

[21] Di Cianni A., Carotenuto A., Brancaccio D., Novellino E., Reubi J.C., Beetschen K., Papini A.M., Ginanneschi M. (2010). Novel octreotide dicarba-analogues with high affinity and different selectivity for somatostatin receptors. J. Med. Chem.

[22] Liu S., Liu Z., Chen K., Yan Y., Watzlowik P., Wester H.J., Chin F.T., Chen X. (2010). 18F-labeled galacto and PEGylated RGD dimers for PET imaging of αvβ3 integrin expression. Mol. Imaging Biol.

[23] Hruby V.J. (2002). Designing peptide receptor agonists and antagonists. Nat. Rev. Drug. Discov.

[24] Rana S., Nissen F., Marr A., Markert A., Altmann A., Mier W., Debus J., Haberkorn U., Askoxylakis V. (2012). Optimization of a novel peptide ligand targeting human carbonic anhydrase IX. PLoS One.

[25] Hilpert K., Winkler D.F., Hancock R.E. (2007). Peptide arrays on cellulose support: SPOT synthesis, a time and cost efficient method for synthesis of large numbers of peptides in a parallel and addressable fashion. Nat. Protoc.

[26] Winkler D.F., Hilpert K., Brandt O., Hancock R.E. (2009). Synthesis of peptide arrays using SPOT technology and the CelluSpots-method. Methods Mol. Biol.

[27] Ahmed S., Mathews A.S., Byeon N., Lavasanifar A., Kaur K. (2010). Peptide arrays for screening cancer specific peptides. Anal. Chem.

[28] Zoller F., Haberkorn U., Mier W. (2011). Miniproteins as phage display-scaffolds for clinical applications. Molecules.

[29] John H., Maronde E., Forssmann W.G., Meyer M., Adermann K. (2008). *N*-terminal acetylation protects glucagon-like peptide GLP-1-(7-34)-amide from DPP-IV-mediated degradation retaining cAMP- and insulin-releasing capacity. Eur. J. Med. Res.

[30] Gentilucci L., de Marco R., Cerisoli L. (2010). Chemical modifications designed to improve peptide stability: incorporation of non-natural amino acids, pseudo-peptide bonds, and cyclization. Curr. Pharm. Des.

[31] Askoxylakis V., Zitzmann-Kolbe S., Zoller F., Altmann A., Markert A., Rana S., Marr A., Mier W., Debus J., Haberkorn U. (2011). Challenges in optimizing a prostate carcinoma binding peptide, identified through the phage display technology. Molecules.

[32] Aloj L., Morelli G. (2004). Design, synthesis and preclinical evaluation of radiolabeled peptides for diagnosis and therapy. Curr. Pharm. Des.

[33] Pnwar P., Iznaga-Escobar N., Mishra P., Srivastava V., Sharma R.K., Chandra R., Mishra A.K. (2005). Radiolabeling and biological evaluation of DOTA-Ph-Al derivative conjugated to anti-EGFR antibody ior egf/r3 for targeted tumor imaging and therapy. Cancer Biol. Ther.

